# Hospital for Epilepsy and Paralysis

**Published:** 1893-05-20

**Authors:** 


### HOSPITAL FOR EPILEPSY AND PARALYSIS,

Portland Terrace, Regent's Park, N.W.

The twenty-sixth annual meeting in support of this hospital was held at the Portman Rooms, Baker Street, on Saturday, May 13th, Sir Massey Lopes in the chair. The Chairman thought the report, which was unanimously adopted, most satisfactory, and said he was glad to note the increased support and interest which was given to the institution. There was matter of congratulation that the hospital was out of debt, a fact which bore evidence to the good management of the committee. He fully approved of the plan of patients paying "according to means," a system which undoubtedly tended to the encouragement of independence and frugality. The committee had to face the fact that they may shortly have to find a new home for the hospital, on account of the Manchester, Sheffield, and Lincolnshire Railway Bill. The Chairman trusted some one would be found generous enough to give a site for a new building when the time came. He then referred to the valuable support of the voluntary system of hospitals given by the report of the Lands' Committee on Metropolitan Hospitals, and alluded to the importance of a uniform system of account. Special hospitals were sometimes found fault with, but they served a very useful purpose, and in his case especially, it would be impossible for patients suffering from such terrible diseases as paralysis and epilepsy to receive the necessary care and attention in a general hospital. Canon Barker said a few cordial words in behalf of the good work done, and the thoughtful solicitude shown to the patients, and went on to condemn the rating of charities as most unjust. He thought not only should the system be abolished, but the House of Commons ought to refund the rates which have already been paid. Short speeches were made by Canon Trench and Colonel Maclagan, and special recognition given to the secretary, Mr. Howgrave Graham, for his valuable services, now extending over more than ten years. Dr. Althaus, in some very interesting remarks upon the medical aspect of the hospital, spoke of the great light thrown upon nervous diseases by recent researches. In mentioning the fact that Jews were peculiarly liable to diseases of this nature, Dr. Althaus said he attributed it to the centuries of persecution through which they had passed. Marvellous operations were now possible, in which it might be said that the knife of the surgeon was guided to the exact spot by the skill of the physician. The meeting concluded with cordial votes of thanks.

